# Geropsychology Training Innovations: Understanding Ageism and Adultism in Students and New Training Initiatives

**DOI:** 10.1093/geroni/igaa057.1995

**Published:** 2020-12-16

**Authors:** Katherine King, Kirsten Graham

## Abstract

In order to address the geropsychology workforce shortage, educators are trying to better understand the attitudes, interests, and needs of prospective students as well as expand educational opportunities and specialty competence. This symposium reports on several recent and ongoing research projects and educational initiatives which can together inform future efforts to recruit students into the field. King et al. discuss how concerns about adultist oppression might keep young adults from entering the field. This study describes a new adultism measure which positively correlates with ageism and negatively correlates with both experience and interest in working with older adults. Together with Strong and Graham’s study looking at ageism among North American and non-North American students, these studies remind us of the continued effort needed to better understand and combat age-related oppression of individuals at both ends of the lifespan. Two studies share specific educational initiatives addressing areas of essential but undervalues areas of training for geropsychologists. O’Malley and Graham report on the integration of health policy training into undergraduate education, while Jacobs et al. describing a new performance-based evaluation tool for training in the assessment of decision-making capacity. Postgraduate specialization continues to be crucial in establishing and growing the discipline of geropsychology. Mlinac and Smith report on the development of a mentorship program for professionals on the path to board certification.

